# Perfused Gills Reveal Fundamental Principles of pH Regulation and Ammonia Homeostasis in the Cephalopod *Octopus vulgaris*

**DOI:** 10.3389/fphys.2017.00162

**Published:** 2017-03-20

**Authors:** Marian Y. Hu, Po-Hsuan Sung, Ying-Jey Guh, Jay-Ron Lee, Pung-Pung Hwang, Dirk Weihrauch, Yung-Che Tseng

**Affiliations:** ^1^Institute of Physiology, University of KielKiel, Germany; ^2^Institute of Cellular and Organismic Biology, Academia SinicaTaipei, Taiwan; ^3^Department of Life Science, National Taiwan UniversityTaipei, Taiwan; ^4^Institute of Biological Chemistry, Academia SinicaTaipei, Taiwan; ^5^Department of Biological Sciences, University of ManitobaWinnipeg, MB, Canada; ^6^Lab of Marine Organismic Physiology, Marine Research Station, Institute of Cellular and Organismic Biology, Academia SinicaTaipei, Taiwan

**Keywords:** NH3/NH4^+^ homeostasis, acid-base regulation, cephalopod, invertebrate, Na^+^/K^+^-ATPase, Rh-protein

## Abstract

In contrast to terrestrial animals most aquatic species can be characterized by relatively higher blood ${\text{NH}}_{4}^{+}$ concentrations despite its potential toxicity to the central nervous system. Although many aquatic species excrete ${\text{NH}}_{4}^{+}$ via specialized epithelia little information is available regarding the mechanistic basis for NH_3_/${\text{NH}}_{4}^{+}$ homeostasis in molluscs. Using perfused gills of *Octopus vulgaris* we studied acid-base regulation and ammonia excretion pathways in this cephalopod species. The octopus gill is capable of regulating ammonia (NH_3_/${\text{NH}}_{4}^{+}$) homeostasis by the accumulation of ammonia at low blood levels (<260 μM) and secretion at blood ammonia concentrations exceeding *in vivo* levels of 300 μM. ${\text{NH}}_{4}^{+}$ transport is sensitive to the adenylyl cyclase inhibitor KH7 indicating that this process is mediated through cAMP-dependent pathways. The perfused octopus gill has substantial pH regulatory abilities during an acidosis, accompanied by an increased secretion of ${\text{NH}}_{4}^{+}$. Immunohistochemical and qPCR analyses revealed tissue specific expression and localization of Na^+^/K^+^-ATPase, V-type H^+^-ATPase, Na^+^/H^+^-exchanger 3, and Rhesus protein in the gill. Using the octopus gill as a molluscan model, our results highlight the coupling of acid-base regulation and nitrogen excretion, which may represent a conserved pH regulatory mechanism across many marine taxa.

## Introduction

Cephalopods including squid, cuttlefish, and octopods have evolved an active lifestyle and vertebrate like sensory abilities to compete with fish for similar resources in marine habitats. Due to their less efficient swimming mode by jet propulsion, locomotion in cephalopods comes at a higher cost, reflected in very high metabolic rates when compared to other active marine animals including sharks and tunas (Rosa and Seibel, 2008). Despite their relatively short lifespan, usually not exceeding 1–2 years, cephalopods have high growth rates and can reach a body mass of several kilograms within 1 year (Llpiński, 2010). This “live fast and die young” lifestyle is predominantly fueled through protein metabolism and ammonia is the main end-product of their amino acid metabolism (Boucher-Rodoni and Mangold, 1988, 1989, 1994). Ammonia in its uncharged gaseous form as NH_3_ can, to a certain extent passively diffuse across biological membranes, whereas the charged ionic form, ${\text{NH}}_{4}^{+}$ cannot cross cell membranes without the help of respective transporters. At physiological pH of ~7.2–7.8, 95–99% of total ammonia occurs in the hydrated form (${\text{NH}}_{4}^{+}$). High concentrations of ammonia are toxic for organisms as they can cause severe detrimental effects on the central nervous system and can lead to intra- as well as extra-cellular acid-base disturbances (Albrecht, 2007). Studies on ammonia tolerance demonstrated LC50-values for environmental ammonia levels in the micromolar range for most aquatic organisms including fish, crustaceans and cephalopods (Miller et al., 1990; Randall and Tsui, 2002; Camargo and Alonso, 2006). Pelagic squids were demonstrated to be particularly sensitive to elevated seawater ammonia concentrations >10 μM (Hanlon, 1990). Thus, potent ammonia excretion pathways must represent an important evolutionary trait of ammonotelic organisms like cephalopods to control intra- and extra-cellular NH_3_/${\text{NH}}_{4}^{+}$ homeostasis.

In most aquatic organisms including fish and crustaceans ammonia is excreted from body fluids into the environment *via* specialized epithelial cells located on the skin or gills (Randall et al., 2004; Tay et al., 2006; Weihrauch et al., 2009; Wright and Wood, 2009; Wu et al., 2010). The current models denote that besides the direct basolateral transport of NH_3_ through Rh-gylcoproteins (e.g., Rhbg) the ammonium ion (${\text{NH}}_{4}^{+}$) may also be imported on the basolateral side *via* the Na^+^/K^+^-ATPase. Hydrated ${\text{NH}}_{4}^{+}$ and K^+^ ions have very similar sizes of ~1.45 Å and due to their K^+^ like behavior ${\text{NH}}_{4}^{+}$ ions can compete with K^+^ as substrates for transporters like the Na^+^/K^+^-ATPase (Skou, 1957; Leone et al., 2014; Quijada-Rodriguez et al., 2015). Alternatively, the basolateral entry of ammonia can be facilitated through NH_3_ channels of the Rh-glycoprotein family that allow the entry and subsequent hydration of NH_3_ to form the ammonium ion in the cytosol. At the apical membrane export of ammonia has been proposed to be facilitated through apical ammonia (NH_3_) channels in combination with Na^+^/H^+^-exchanger (NHE) or proton pump-based [e.g., V-type H^+^-ATPase (VHA), H^+^/K^+^-ATPase] H^+^ extrusion mechanisms that lead to an acid trapping of NH_3_ that has been excreted through the NH_3_ channels, in the outer boundary layer of excretory epithelia (Wright and Wood, 2009; Gruswitz et al., 2010; Nawata et al., 2010; Wu et al., 2010). The removal of the proton from the ammonium ion at the apical membrane is potentially facilitated by the deprotonating activity found in NH_3_ channel proteins (Javelle et al., 2008). Besides ammonia excretion mechanisms involving transporters, an alternative model has been proposed for the gills of the green shore crab *Carcinus maenas*, where ${\text{NH}}_{4}^{+}$ excretion has been demonstrated to be highly sensitive to the microtubule inhibitors, colchicine, taxol and thiabendazole, suggesting a vesicular ammonia (NH_3_) trapping in acidified vesicles and subsequent transport of the potentially toxic ${\text{NH}}_{4}^{+}$ ions across the cytosol (Weihrauch et al., 2002).

Similar to the situation in fish and crustaceans the gills of cephalopods are probably also the most important sites for gas exchange and pH regulation (Hu et al., 2010, 2011, 2014b). Earlier studies suggested that despite the existence of other potential excretory organs such as branchial hearts or renal appendages (equivalent to the vertebrate kidneys) gills may represent the most important site for ${\text{NH}}_{4}^{+}$ excretion in cephalopods (Potts, 1965). High concentrations of Na^+^/K^+^-ATPase localized in basolateral membranes of branchial epithelia of cephalopods underline their predominant role in active ion transport and excretion (Schipp et al., 1979; Hu et al., 2011). Furthermore, the expression of transporters and channels like the teleost orthologs Na^+^/H^+^-exchanger 3 (NHE3) and Rh-proteins (RhP) localized in apical membranes suggest an involvement of gill epithelia in acid-base balance and ammonia transport in cephalopods (Hu et al., 2014b). Recent studies conducted on squid *Sepioteuthis lessoniana*, have demonstrated that acidified seawater stimulates the expression of branchial acid-base transporters including V-type H^+^-ATPase, Na^+^/${\text{HCO}}_{3}^{-}$-cotransporter (NBC), NHE3, and one primitive Rh-protein (RhP) suggesting that pH regulation and ${\text{NH}}_{4}^{+}$ excretion are coupled processes (Hu et al., 2014b).

To date, ammonia excretion mechanisms are still poorly understood for most invertebrate species. Although, some information is available for crustaceans (reviewed in Henry et al., 2012), ammonia excretion mechanisms are virtually unexplored for the entire phylum mollusca. Thus, the exclusively ammonotelic nature of cephalopods and their extensive protein metabolism makes this taxonomic group an interesting specimen to investigate ${\text{NH}}_{4}^{+}$ excretion pathways.

Gill perfusion experiments were successfully applied in aquatic organisms including fish and crustaceans allowing the examination of transport rates and metabolic demands in an isolated organ (Tresguerres et al., 2008; Deigweiher et al., 2010; Fehsenfeld and Weihrauch, 2013). Using perfused gills of the common octopus, *Octopus vulgaris*, the present work aims to demonstrate that pH regulation and ${\text{NH}}_{4}^{+}$ transport are coupled processes in complex branchial epithelia of this cephalopod species. The present work introduces a new molluscan model to study acid-base regulation and ${\text{NH}}_{4}^{+}$ excretion mechanisms. This will help understanding nitrogen excretion pathways in cephalopods, a taxonomic group that has received little attention regarding ionic regulation associated with extracellular pH and ammonia homeostasis.

## Materials and methods

### Experimental animals

Experimental animals, *O. vulgaris*, were obtained from a local dealer in Keelung, Taiwan, and transported in well aerated containers to the Marine Research Station, Institute of Cellular and Organismic Biology, at the Academia Sinica (I-lan, Taiwan). Animals ranging from 300 to 600 g were held in closed circulatory seawater systems of ~600 l for several days before they were used for experimental procedures. Individuals were kept separately in cages at a constant 12/12 h light cycle at 26°C and low seawater ammonia levels <5.6 μmol l^−1^ and were fed with live clams (*Meretrix* spp.). For experimental procedures including perfusion experiments and blood sampling, animals were anesthetized by cooling below 5°C until full depression of ventilation and were killed by decapitation. Best practices for handling cephalopods as experimental animals were followed, including currently discussed ethical standards for anesthesia and killing of cephalopods (Andrews et al., 2013). The experimental protocols were approved by the Academia Sinica Institutional Animal Care and Utilization Committee (approval no. RFIZOOHP220782).

### Determination of blood pH and ${\text{NH}}_{4}^{+}$ concentrations

Blood samples were collected from the *vena cava via* a gas-tight Hamilton syringe by dissecting the funnel and mantle from the ventral side. Determination of extracellular pH (pH_*e*_) and ${\text{NH}}_{4}^{+}$ were conducted as previously described (also see Supplementary Information for Materials and Methods) using a WTW 340 pH meter (precision ± 0.01 units) equipped with a microelectrode (WTW Mic-D). The pH meter was calibrated with Radiometer precision buffers 7 and 10 (S11M44, S11 M007) and pH-values are provided using the NBS (National Bureau of Standards) scale.

### Perfusion experiments

For perfusion experiments gills were carefully dissected from the mantle along the branchial gland, and two cuts at the 1st order afferent and efferent vessels were made before the gill was carefully detached from the mantle and intestinal sac. All dissection procedures were conducted while the tissues were immersed in seawater. Five centimeters of long polyethylene tubes with an outer diameter of 1.52 mm and an inner diameter of 0.86 mm that were tapered toward the end, were inserted into the 1st order efferent and afferent vessels of the octopus gill. Synthetic woven threads of 0.2 mm diameter were used to tie branchial vessels to the PVC tubes. PVC tubes were connected to thicker (2 mm) silicon tubes and the afferent tube was connected to a peristaltic pump, pumping perfusion saline adjusted to 990 ± 10 mOsm Kg^−1^ (see Supplemental Table 1 for saline composition) at a rate of 12 ml h^−1^ through the gill. Furthermore, ionic composition of *Octopus* blood was analyzed to validate the composition of our artificial octopus blood (Supplemental Table 1). And the ionic composition of the saline is close to that of natural seawater as seawater has been demonstrated to be a suitable Ringer's solution for most invertebrates (Robertson, 1949). Before each experiment the pH of the saline was adjusted by addition of HCl or NaOH. The Na^+^, K^+^, and Ca^2+^ were measured using an atomic absorption spectrophotometer (Hitachi Z-8000, Tokyo, Japan). And Cl^−^ content was measured using a double-beam spectrophotometer (NanoDrop 2000/2000c UV-Vis Spectrophotometer, Thermo Scientific). For this 500 μl of diluted samples were mixed with 500 μL of solution containing Hg(SCN)_2_ (0.3 g in 95% ethanol) and NH_4_Fe(SO_4_)_2_ 12H_2_O (30 g in 135 ml 6 N HNO_3_). Standard solutions of major ions (Na^+^, K^+^, Ca^2+^, and Cl^−^; Merck, Darmstadt, Germany) were used to generate standard curves. In addition, ${\text{HCO}}_{3}^{-}$ content was determined from total dissolved carbon measurements using a Corning 965 carbon dioxide analyzer (Olympic Analytical Service, England) as previously described (Hu et al., 2014b, 2016). At the efferent vessel of the gill perfusion saline was drained through a PVC tube into a collecting vessel. The entire gill was immersed in an aerated bathing solution (same as perfusion saline, without addition of ammonia) in a volume of 50 ml at 25°C that continuously irrigated the gill with oxygenated saline (see Figure S1 for a schematic illustration of the setup).

Before the start of experiments using perfusion salines with different NH_3_/${\text{NH}}_{4}^{+}$ concentrations (prepared by the addition of NH_4_Cl to perfusion sline), pH (adjusted with HCL and NaOH) or pharmacological compounds (e.g., 10 μM KH7 and 75 μM cAMP) gills were perfused with the respective saline for 10 min to achieve full exchange of fluids within the tubes and gill vessels. 8-Bromoadenosine 3′,5′-cyclic monophosphate (cAMP) was dissolved in perfusion saline to a final concentration of 75 μmol l^−1^ and a stock solution of the specific soluble adenylate cyclase (sAC) inhibitor KH7 (Bitterman et al., 2013) was dissolved in DMSO and was diluted to 10 μmol l^−1^ with <0.1% DMSO in the final perfusion saline. The duration of perfusion experiments never exceeded 1.5 h to assure full vitality of the examined tissues.

### Immunohistological staining

Immunohistochemical analyses of acid-base transporters in *O. vulgaris* gills were conducted as previously described (Hu et al., 2010, 2014b). Gill tissues were fixed by direct immersion in Bouin's fixative followed by rinses in 75% ethanol. Samples were embedded in Paraplast (Paraplast Plus, Sigma, P3683) and sections of 4 μm were cut on a Leica RM2265 microtome. The slides were deparaffinized in Histoclear II® for 10 min and passed through a descending alcohol series and transferred to a PBS solution containing 5% bovine serum albumin (BSA) for 30 min to block non-specific binding. The primary antibodies, a rabbit polyclonal antibody H-300, raised against the human α subunit of the Na^+^/K^+^-ATPase (NKA) (Santa Cruz Biotechnology, INC) and custom made polyclonal antibodies raised against part of the carboxyl-terminal region (IYRVRKVGYDEQFIMSY) of Na^+^/H^+^-exchanger3 (NHE3), the subunit A region (SYSKYTRALDEFYDK) of the V-type-H^+^-ATPase (VHA), and the Rhesus protein (RhP) of squid, *S. lessoniana* (antibody designed against the synthetic peptide TRAGYQEFKW) were diluted in PBS (1:100) and placed in small droplets of 200 μl onto the sections, and incubated for 12 h at 4°C in a wet chamber. For the peptide competition assay (PCA), primary antibodies were pre-absorbed by their specific peptides at a concentration of 0.1 mg/ml for 8 h at 4°C. For detailed information regarding the antibodies used refer to Hu et al. (2010, 2014b). The sections were then washed in PBS and incubated for 1 h with small droplets (200 μl) of secondary antibody, anti-mouse Alexa Fluor 488 or anti- rabbit Alexa Fluor 568 (Invitrogen) (dilution 1:250). To allow double-color immunofluorescence staining, one of the polyclonal antibodies was directly labeled with Alexa Fluor dyes using the Zenon antibody labeling kit (Molecular Probes, Eugene, OR, USA). After rinses in PBS, sections were examined with a fluorescence microscope (Zeiss imager A1) equipped with an appropriate filter set.

### Molecular cloning

Acid-base transporters' peptide sequences from cephalopods and other species (aquatic animals were given the highest priority) were used to BLAST the *O. vulgaris* expressed sequence tag (EST) and octopus genome databases in NCBI. Based on those putative sequences assembled from collected tags, gene specific primers (listed in Supplemental Table 2) were designed for the reverse transcription polymerase chain reaction (RT-PCR) analysis. Detailed cloning procedures and further phylogenetic analysis are presented in the Supplementary Information for Material and Methods.

### Real-time quantitative PCR (qPCR)

The mRNA expressions of target genes were measured by qPCR with the Roche LightCycler® 480 System (Roche Applied Science, Mannheim, Germany). The sequence accession numbers and primers are depicted in Supplemental Table 3. qPCR assay procedures are described in the Supplementary Information for Materials and Methods.

### Statistical analyses

Statistical analyses were performed using Sigma Stat 3.0 (Systat) software. Statistical differences between blood pH and ${\text{NH}}_{4}^{+}$ levels at different transit stations as well as the effects of cAMP, KH7 and pH on ${\text{NH}}_{4}^{+}$ excretion rates were analyzed by one-way ANOA followed by Tukeys *post-hoc* test. A Student's *t*-test was used to compare blood pH and ${\text{NH}}_{4}^{+}$ concentrations before and after gill passage in perfusion experiments. Two-way ANOVA was used for analyzing differences between H^+^ loss and ${\text{NH}}_{4}^{+}$ excretion rates with perfusion salines of different pH and ${\text{NH}}_{4}^{+}$ concentrations. Data sets were normally distributed (Kolmogorov-Smirnov test). Equal variance was tested using the Levene median test. The significance levels were set to *p* < 0.05 and 0.01.

## Results

### Regulation of pH and ${\text{NH}}_{4}^{+}$ homeostasis by branchial epithelia

**Figure 1:**
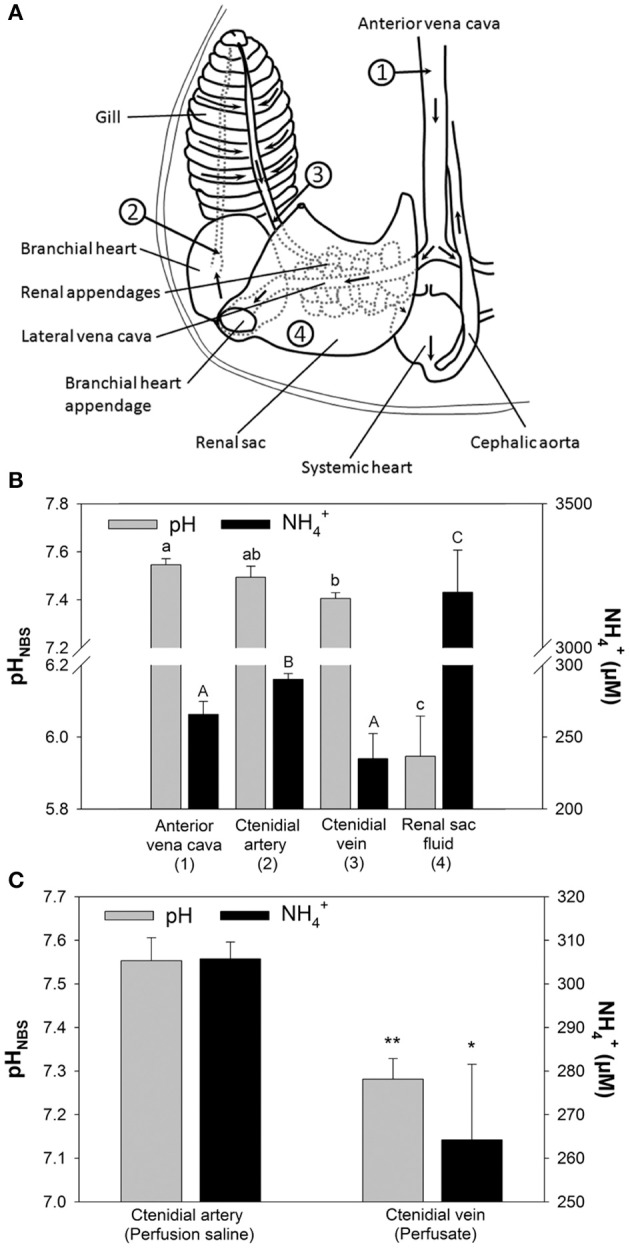
**Determination of pH and ${\text{NH}}_{4}^{+}$ concentrations in perfused ***Octopus vulgaris*** gills. (A)** Transit stations before and after passage of excretory organs including renal appendages, branchial heart appendages, and gills. Numbers indicate the sampling stations for *in vivo* measurements of blood pH and ${\text{NH}}_{4}^{+}$ concentrations shown in **(B)** (Figure modified after Potts, 1965). **(B)** *In vivo* measurements for pH and ${\text{NH}}_{4}^{+}$ concentrations at different transit stations before and after passage of major excretory organs. **(C)** pH and ${\text{NH}}_{4}^{+}$ levels before (perfusion saline) and after gill passage (perfusate) in perfused gills. Values are presented as mean ± *SE* (*n* = 4–5) and capital letters denote significant differences between pH measurements whereas lower case letters denote significant differences between ${\text{NH}}_{4}^{+}$ measurements along the passage of excretory organs. Asterisks indicate significant differences of pH and ${\text{NH}}_{4}^{+}$ levels before and after gill passage in perfusion experiments.

The major excretory organs of *O. vulgaris*, such as gills and renal appendages, are connected by blood vessels that direct the blood flow from the vena cava through these organs to the systemic heart (Figure 1A). Blood returning from the body through the *vena cava* (station 1) enters the lateral vena cava and is distributed to the renal appendages from where the urine is secreted into the renal sac (station 4). After passage of the renal appendages the blood flow reaches the branchial heart by passing the branchial heart appendages. From here the blood is pumped through the gill *via* the 1st order afferent vessels (station 2) and leaves the gill *via* the efferent 1st order vessels (station 3) from where it enters the systemic heart. From here the oxygenated blood is pumped back into the body.

Sampling of blood from different blood transit stations before and after passage through major excretory organs, including gills and renal appendages, demonstrated changes in blood ${\text{NH}}_{4}^{+}$ concentrations and pH (Figures 1A,B). Blood pH-values in *O. vulgaris* ranged from pH 7.4 to 7.6 depending on the sampling site. Determination of blood pH at different transit stations demonstrated a decrease in pH by ~0.1 pH units between blood samples from the vena cava (station 1) and after gill passage (station 3). Blood ${\text{NH}}_{4}^{+}$ levels ranged from 240 to 300 μM, depending on the sampling site. ${\text{NH}}_{4}^{+}$ concentrations were significantly decreased by 60 μM after gill passage (station 3) compared to blood samples taken from the afferent vessel (station 2). The renal sac fluid (urine) was highly acidic with a pH-values being as low as 5.8 and is characterized by very high ${\text{NH}}_{4}^{+}$ concentrations of 3,192 ± 146 μM. Determination of pH and ${\text{NH}}_{4}^{+}$ concentrations before and after gill passage in perfused gills could demonstrate a drop in pH by 0.2 pH units associated with a drop in perfusion saline ${\text{NH}}_{4}^{+}$ levels of 40 μM (Figure 1C). The stability of this decrease in pH after gill passage served as a reliable indication for the viability of the perfused gill. During perfusion experiments exceeding 2 h the difference in pH between saline before and after gill passage decreased indicating a progressively reduced viability of gill tissues.

**Figure 2:**
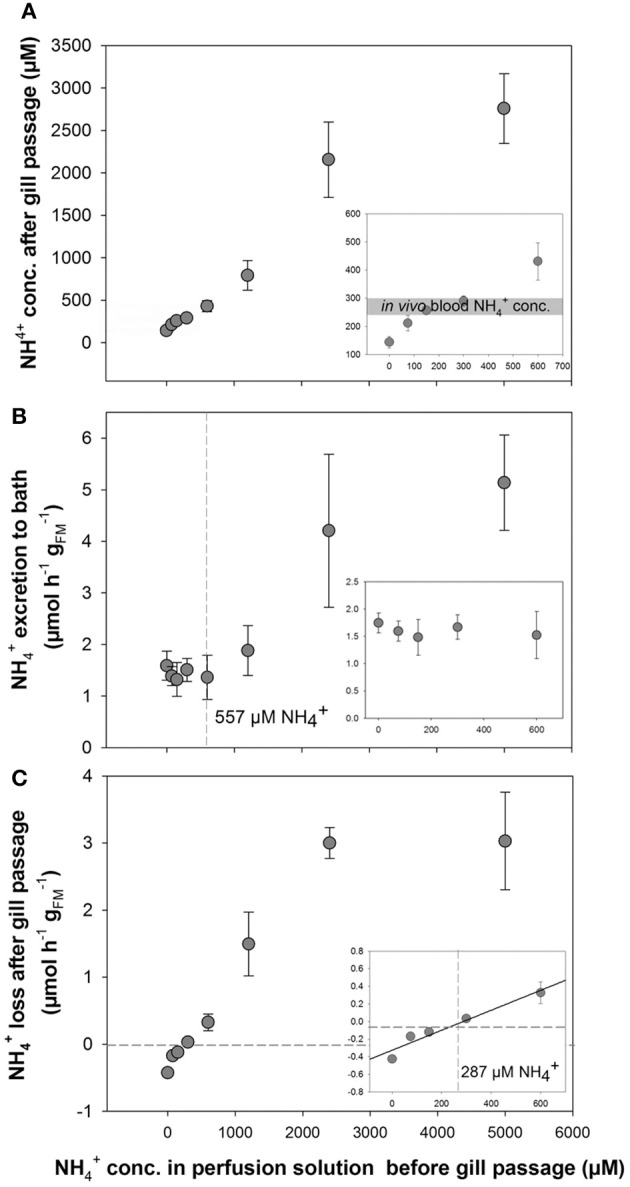
**Determination of ${\text{NH}}_{4}^{+}$ transport in perfused ***Octopus vulgaris*** gills. (A)** ${\text{NH}}_{4}^{+}$ concentrations in the perfusate after gill passage as a function of different blood ${\text{NH}}_{4}^{+}$ concentrations ranging from 0 to 5,000 μM. Insert showing the natural range of *in vivo* blood ${\text{NH}}_{4}^{+}$ concentrations (gray bar) in the range of 0–600 μM ${\text{NH}}_{4}^{+}$. **(B)** ${\text{NH}}_{4}^{+}$ excretion rates (presented as μmol ${\text{NH}}_{4}^{+}$ h^−1^ ${\text{g}}_{\text{FM}}^{-1}$; fresh mass: FM) in perfused gills measured in the bath as a function of blood ${\text{NH}}_{4}^{+}$ levels up to 5,000 μM (Insert: ${\text{NH}}_{4}^{+}$ excretion rates at blood ${\text{NH}}_{4}^{+}$ concentrations ranging from 0 to 600 μM). The dashed line indicates the blood ${\text{NH}}_{4}^{+}$ concentration where ${\text{NH}}_{4}^{+}$ starts to be excreted into the bath. **(C)** Transport rates of ${\text{NH}}_{4}^{+}$ from the perfusion saline after gill passage as a function of blood ${\text{NH}}_{4}^{+}$ levels. Note the negative transport rates below blood ${\text{NH}}_{4}^{+}$ levels of 287 μM indicated by dashed lines (inset: ammonia transport rates at blood ${\text{NH}}_{4}^{+}$ concentrations ranging from 0 to 600 μM). Values are given as mean ± *SE* (*n* = 4–7).

Perfusion experiments using artificial blood salines with different ${\text{NH}}_{4}^{+}$ concentrations ranging from 0 to 5,000 μM were used to test the ${\text{NH}}_{4}^{+}$ regulatory capacity of *O. vulgaris* gills. Surprisingly, an enrichment of ammonia was observed in the post branchial fluid at initial ${\text{NH}}_{4}^{+}$ concentrations below 300 μM, whereas at higher concentrations a loss of ammonia was detected in the post-branchial fluids (Figure 2A). This reduction in blood ${\text{NH}}_{4}^{+}$ was most evident at 5,000 μM with only 2,700 μM ${\text{NH}}_{4}^{+}$ remaining after a single gill passage leading to a non-linear flattened curve using blood salines with ${\text{NH}}_{4}^{+}$ concentrations >300 μM (Figure 2A). This bi-phasic regulation was also evident when looking at ${\text{NH}}_{4}^{+}$ excretion rates into the bathing saline where ${\text{NH}}_{4}^{+}$ excretion was maintained at a constant rate of ~1.5 μmol ${\text{NH}}_{4}^{+}$ h^−1^ ${\text{g}}_{\text{FM}}^{-1}$ at blood ${\text{NH}}_{4}^{+}$ concentrations between 0 and 600 μM (Figure 2B). At blood (perfusion solution) ${\text{NH}}_{4}^{+}$ concentrations >600 μM an increased excretion rate was observed with 1.89 ± 0.49, 4.21 ± 1.48, and 5.14 ± 0.93 μmol ${\text{NH}}_{4}^{+}$ h^−1^ ${\text{g}}_{\text{FM}}^{-1}$ at initial perfusion solution ${\text{NH}}_{4}^{+}$ concentrations of 1,200, 2,500, and 5,000 μM, respectively.

A gain of ammonia was observed in the perfusate, when gills were perfused with solutions initially containing ${\text{NH}}_{4}^{+}$ concentrations below 300 μM (Figures 2A,C). At 300 μM or higher ${\text{NH}}_{4}^{+}$ concentrations in the initial perfusion solution a positive rate of ${\text{NH}}_{4}^{+}$ loss from the blood was observed that increased in a linear fashion as a function of blood ${\text{NH}}_{4}^{+}$ levels up to 2,400 μM. At a blood ${\text{NH}}_{4}^{+}$ concentration of 5,000 μM the rate of ${\text{NH}}_{4}^{+}$ removal from the blood after gill passage was not further increased but was maintained at a rate of ~3 μmol ${\text{NH}}_{4}^{+}$ h^−1^ ${\text{g}}_{\text{FM}}^{-1}$ similar to excretion rates determined for 2,400 μM ${\text{NH}}_{4}^{+}$. A linear regression analysis for blood ${\text{NH}}_{4}^{+}$ concentrations ranging from 0 to 600 μm shows the blood ${\text{NH}}_{4}^{+}$ concentration of 287 μM at which no net excretion of ${\text{NH}}_{4}^{+}$ was measured (Figure 2C, inset).

### Effects of cAMP, KH7, and pH on branchial ammonia regulation

**Figure 3:**
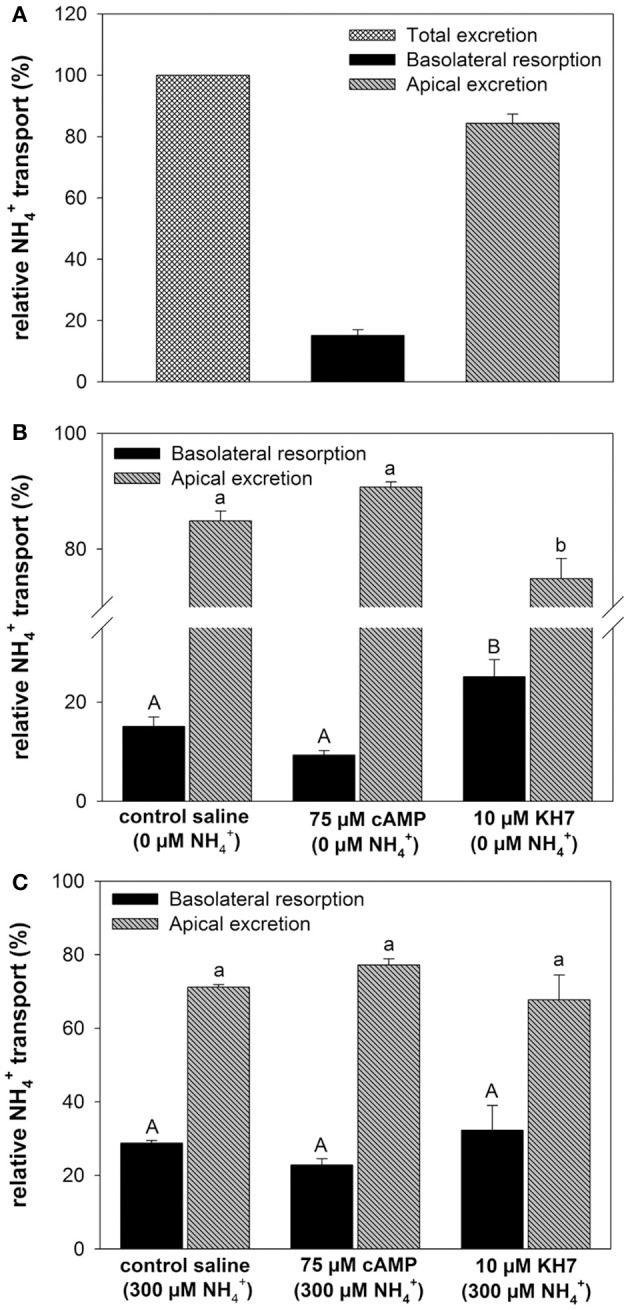
**Effects of cAMP and KH7 on ${\text{NH}}_{4}^{+}$ transport in perfused cephalopod gills. (A)** Relative proportion of ${\text{NH}}_{4}^{+}$ transported to the bath (apical) and to the perfusate (basolateral) in perfused gills at a blood ammonia of 0 μM. **(B)** Effects of cAMP and KH7 on branchial ammonia transport rates at 0 μM blood ${\text{NH}}_{4}^{+}$ (One-way ANOVA *df* = 2.4; *F* = 19.565; *p* < 0.001) and 300 μM ${\text{NH}}_{4}^{+}$ **(C)**. Transport rates are divided into apical secretion to the bath (black) and basolateral resorption of ${\text{NH}}_{4}^{+}$ into the perfusate after gill passage. Values are presented as mean ± *SE* (*n* = 4–5) and capital letters denote significant differences between basolateral resorption rates whereas lower case letters denote significant differences between apical ${\text{NH}}_{4}^{+}$ excretion rates.

At a blood ${\text{NH}}_{4}^{+}$ concentration of 0 μM the metabolically produced ${\text{NH}}_{4}^{+}$ by the gill led to a total excretion of 4.55 ± 1.73 μmol ${\text{NH}}_{4}^{+}$ h^−1^ ${\text{g}}_{\text{FM}}^{-1}$ of which 84% were excreted across the apical side into the bath and 16% being transported into the blood (Figure 3A). Addition of cAMP had no significant effect (*p* = 0.098) on relative ${\text{NH}}_{4}^{+}$ transport rates toward both, the apical and the basolateral side regardless whether initial perfusate ${\text{NH}}_{4}^{+}$ concentrations were set to 0 and 300 μM (Figures 3B,C). However, the specific soluble adenylyl cyclase (sAC) inhibitor KH7 significantly (One-way ANOVA *df* : 2.4; *F*: 19.565; *p* < 0.001, *Post-hoc* test *p* = 0.034) decreased apical ${\text{NH}}_{4}^{+}$ excretion rates into the bath and increased basolateral secretion of ${\text{NH}}_{4}^{+}$ into the blood at blood ${\text{NH}}_{4}^{+}$ concentrations of 0 μM (Figure 3B). No effects of KH7 were measured at an initial blood ${\text{NH}}_{4}^{+}$ concentration of 300 μM (Figure 3C).

**Figure 4:**
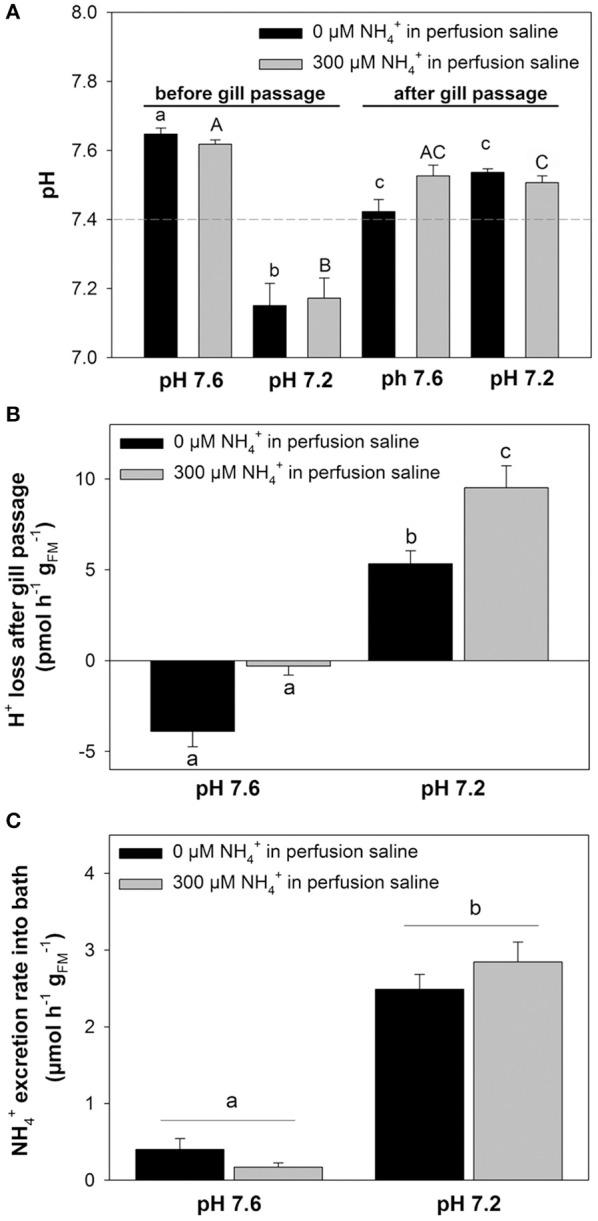
**Coupling of ${\text{NH}}_{4}^{+}$ excretion and pH regulation in perfused octopus gills. (A)** pH regulatory abilities of perfused gills at two different (0 and 300 μM) blood/perfusion saline ${\text{NH}}_{4}^{+}$ concentrations. Perfusion salines were adjusted to two different pH levels, 7.6 and 7.2 shown on the x-axis and the two ammonia levels separated by black and gray bars. Actual blood/perfusion saline pH levels before and after gill passage are shown on the y-axis. The dashed line indicates the average *in vivo* pH of arterial blood in *O. vulgaris*. **(B)** Determination of proton secretion rates by the gill from the difference in [H^+^] before and after gill passage at two different pH levels and ${\text{NH}}_{4}^{+}$ concentrations, respectively. **(C)** ${\text{NH}}_{4}^{+}$ excretion rates of perfused gills using pH 7.6 and pH 7.2 salines at 0 and 300 μM blood ammonia levels. Values are presented as mean ± *SE* (*n* = 4–5) and letters denote significant differences between pH and ${\text{NH}}_{4}^{+}$ treatments.

In a second set of experiments we investigated the effects of an extracellular acidosis (pH 7.2) on the branchial pH regulatory capacities and epithelial ${\text{NH}}_{4}^{+}$ transport (Figure 4). The effects of an acidosis were combined with the initial blood ${\text{NH}}_{4}^{+}$ concentrations of 0 and 300 μM, respectively. The pH levels for the two ${\text{NH}}_{4}^{+}$ concentrations before gill passage were compared to those after gill passage. At an initial (before gill passage) blood pH of ~7.6 a significant decrease in blood pH after gill passage was observed for 0 μM ${\text{NH}}_{4}^{+}$ (Figure 4A). Under initial conditions of ~pH 7.2, an elevation of blood pH back to control levels ranging between pH 7.4 and 7.5 was measured for both blood ${\text{NH}}_{4}^{+}$ concentrations, 0 and 300 μM (Figure 4A). This compensation reaction toward an initially induced acidosis was also indicated by a significantly increased transport rate of protons from the blood during gill passage (Figure 4B). Here an increase in transport rate of H^+^ from the blood was measured for blood containing initially 300 μM ${\text{NH}}_{4}^{+}$ (9.52 ± 1.21 pmol h^−1^ ${\text{g}}_{\text{FM}}^{-1}$) compared to blood containing 0 μM ${\text{NH}}_{4}^{+}$ (5.33 ± 0.72 pmol h^−1^ ${\text{g}}_{\text{FM}}^{-1}$). A higher rate of proton removal from the blood under acidified conditions was accompanied by increased ${\text{NH}}_{4}^{+}$ excretion rates compared to control (pH 7.6) conditions (Figure 4C). Compared to control saline of pH 7.6 ${\text{NH}}_{4}^{+}$ excretion rates increased by 6-fold and by 16-fold when perfused with acidified saline of 7.2 initially containing either 0 or 300 μM ${\text{NH}}_{4}^{+}$.

### Tissue expressions of VHA, NHE3, NKA, and RhP

**Figure 5:**
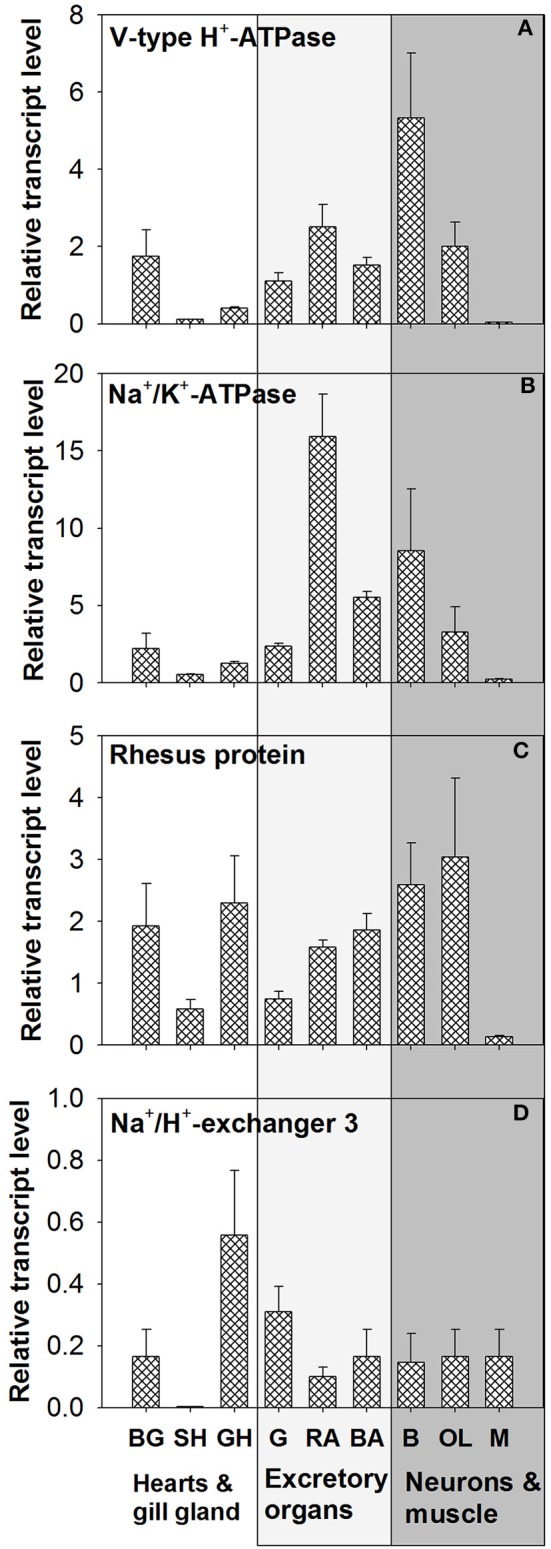
**Tissue specific expression of acid-base transporters**. Expression profiles of **(A)** VHA, **(B)** NKA, **(C)** RhP, and **(D)** NHE3 in various tissues determined by qPCR analysis (for tissue panel see Figure S2). The different tissues were classified into three functional groups circulatory system (hearts and branchial gland), excretory organs, and neurons and muscles. BG, branchial gland; SH, systemic heart; GH, gill heart; G, gill; RA, renal appendages; BA, branchial heart appendages; B, brain; OL, optical lobe; M, muscle.

Tissue expression levels of VHA demonstrated highest abundance of transcripts in brain (B) tissues (Figure 5A and Figure S2). Relatively similar mRNA expression levels were detected in gills, renal appendages (RA), branchial heart appendages (BA), and optical lobes (OL). Low expression levels of VHA were determined for systemic heart (SH), gill hearts (GH), and mantle (M) tissues. Statistical analyses demonstrated significant differences in the expression level between SH vs. GH; *p* = 0.04, SH vs. OL; *p* = 0.03 and between GH vs. M; *p* = 0.04). PCR as well as qPCR analyses demonstrated highest NKA expression levels in RA, followed by neurons (B + OL) and other excretory organs including gills (G) and (BA) (Figure 5B). Statistical analyses demonstrated significant differences in the expression level between SH vs. G; *p* = 0.04, SH vs. BHA; *p* = 0.04 and between BHA vs. M; *p* = 0.03). Expression levels of RhP are highest in neurons (B + OL) followed by tissues of the circulatory system including branchial gland (BG) and excretory organs including RA, BA, and G (Figure 5C). Lowest transcript levels of RhP were detected in SH and M tissues. Statistical analyses demonstrated significant differences in the expression level between RA vs. M; *p* = 0.04). Highest expression levels for NHE3 that belongs to the invertebrate clade of NHE3 (see Figure S3 for phylogenetic analysis) were detected in GH and G, whereas the remaining tissues had relatively similar expression levels (Figure 5D). Very low expression levels for this transporter were measured in the systemic heart (SH).

### Gill morphology and localization of branchial acid-base transporters

**Figure 6:**
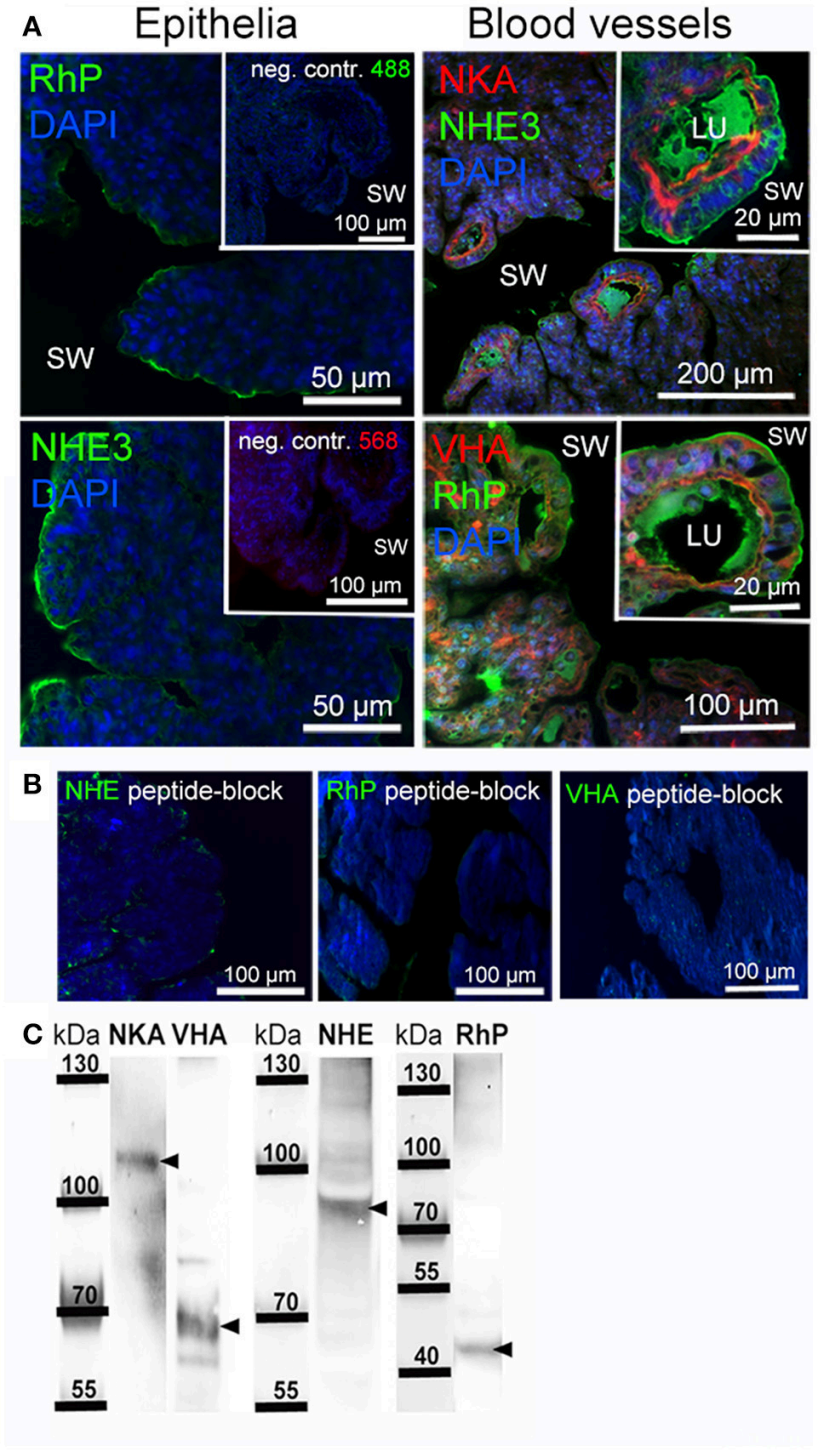
**Localization of branchial acid-base and NH_3_ transporters. (A)** Positive immunoreactivity of important transporters was mainly observed in blood vessels and epithelial cells. The NKA is located in basolateral membranes and the VHA antibody shows positive immunoreactivity in basolateral membranes, the cytosol as well as endothelial cells of blood vessels. Positive NHE3 and RhP immunoreactivity is mainly restricted to apical membranes of blood vessels and branchial epithelia facing the seawater. Autofluorescence of the blood was observed in blood vessels of the gill using 488 nm excitation and the 530 nm emission filter. RhP and NHE immunoreactivity is abolished by incubating the primary antibodies with their specific peptides for 8 h. **(B)** Negative controls by omitting the primary antibody demonstrate no unspecific binding of the secondary antibodies used. **(C)** Western blot analysis of antibodies used, indicating specific immunoreactivity with proteins in the predicted size range (indicated by arrows). LU, blood vessel lumen; sw, sea water.

Immunohistochemical analyses were used to describe the morphology of the octopus gill and to identify epithelia that are rich in acid-base transporters relevant for pH and ${\text{NH}}_{4}^{+}$ regulation (Figure 6A). The morphology of the octopus gill differs from that of most cephalopods (e.g., squid and cuttlefish) by having a less organized hierarchy in folding pattern of 1st to 3rd order lamellae. Instead of continuous fan like folds as found in squid and cuttlefish the 2nd order lamellae of octopods are joined and the 3rd order lamellae are branching folds (Figure 6A). Immunohistochemical stainings demonstrated a predominant signal of the NKA in the blood vessels with a predominant basolateral localization in membranes of the outer epithelium. While some larger blood vessels face the seawater, others are located within the gill. All blood vessels branch out toward the periphery of the gill where the blood is merely separated by the thin branchial epithelium from the surrounding seawater. Additionally, branchial blood vessels show positive immunoreactivity for VHA. The VHA antibody shows weaker immunoreactivity in basolateral membranes compared to the NKA but shows a distinct localization of the VHA in the cytosol of endothelial cells of blood vessels. However, this antibody shows a strong signal in endothelial cells lining the inner side of the blood vessel (Figure 6A). Distinct NHE3 and RhP immunoreactivity was observed in apical membranes of blood vessel epithelial cells. Additionally, endothelial cells facing the lumen of the blood vessel also show a weak immunoreactivity of NHE3 and RhP in basolateral membranes. Besides expression of NHE3 and RhP in blood vessels, a distinct positive immuno-reactivity was also found in apical membranes of gill epithelial cells facing the seawater. Peptide compensation assays using antibody-specific peptides demonstrated full abolishment of the immunoreactivity on gill section (Figure 6B). Western blot analysis demonstrated distinct immunoreactivity with proteins in the predicted size range (Figure 6C).

## Discussion

### Changes in blood pH and ${\text{NH}}_{4}^{+}$ during passage of excretory organs

*In vivo* determinations of *O. vulgaris* blood pH and NH_3_/${\text{NH}}_{4}^{+}$ are in general in accordance with earlier findings by Potts (1965), demonstrating that during passage through excretory organs, including renal appendages, branchial heart appendages and gills, the majority of ${\text{NH}}_{4}^{+}$ is excreted across gill epithelia. The passage of blood through excretory organs is accompanied by a drop in blood pH-values. In contrast, determinations of blood pH during gill passage using implemented catheters demonstrated an increase in blood pH after gill passage in the free swimming octopus (Houlihan et al., 1986). These different findings may be explained by the different sampling methods in anesthetized and free swimming animals and requires further clarification. In the anesthetized animals a slight but significant increase in blood ${\text{NH}}_{4}^{+}$ level was observed during blood passage through renal appendages. The renal appendages are enclosed by the renal sacs, filled with an acidic (~pH 6) urine that contains high concentrations of ammonia (~3.2 mM). Despite potential ammonia trapping in this acidic urine, a small amount of NH_3_/${\text{NH}}_{4}^{+}$ may diffuse back into the blood (at regular blood ${\text{NH}}_{4}^{+}$ levels below 300 μM) across epithelia of the renal appendages. This would explain the increase in blood ${\text{NH}}_{4}^{+}$ levels after passage of excretory organs within the renal sacs. *Ex vivo* measurements of pH and ${\text{NH}}_{4}^{+}$ concentrations within the artificial blood before and after passage through the isolated perfused gill were in accordance to observations made in the intact animal, demonstrating that the isolated gill behaves in a similar fashion compared to *in vivo* conditions. Furthermore, contractile movements of the gill supported perfusion of the gill and demonstrated the viability of the isolated perfused gill under *ex vivo* conditions for up to 2 h. In other aquatic species, isolated gill tissues showed viability and functionality over a time span of several hours. For instance, long-term recordings of oxygen consumption and ion transport capacities in isolated fish gill preparations demonstrated the viability of the perfused organ for at least 1–2 h and for the gills of the blue crab *Callinectes sapidus* 4 h, respectively (Perry et al., 1984; Burnett and Towle, 1990; Deigweiher et al., 2010).

### Ammonia transport in perfused gills of *Octopus vulgaris*

In order to test the transport properties of ammonia across the gill epithelium we perfused gills with artificial blood containing different concentrations of ${\text{NH}}_{4}^{+}$, ranging from 0 to 5,000 μM. In response to an ammonia load exceeding *in vivo* blood ${\text{NH}}_{4}^{+}$ concentrations (≈300 μM) the *octopus* gill showed an outward directed net transport of ${\text{NH}}_{4}^{+}$. This finding is in general accordance with observations made in other aquatic organisms including fish and crustaceans (reviewed by Weihrauch et al., 2009). The magnitude of ${\text{NH}}_{4}^{+}$ excretion rates across gill epithelia are in accordance to findings in crustaceans and fish. For example, the green shore crab *C. maenas* has branchial ${\text{NH}}_{4}^{+}$ excretion rates of ca. 35 μmol h^−1^ ${\text{g}}_{\text{FM}}^{-1}$ when gills were perfused with hemolymph-like salines containing 200 μM NH_4_Cl and excretion rates of 10–20 μmol h^−1^ ${\text{g}}_{\text{FM}}^{-1}$ when symmetrical bath and perfusion salines with ${\text{NH}}_{4}^{+}$ concentrations of 100 μM were applied (Weihrauch et al., 1998, 2002). Ammonia excretion rates of perfused gills of marine teleosts demonstrated excretion rates of 0.18–0.3 μmol h^−1^ ${\text{g}}_{\text{FM}}^{-1}$ at relatively high blood ${\text{NH}}_{4}^{+}$ concentrations of 1 mM (Goldstein et al., 1982). Interestingly, our results on the octopus gill demonstrated that below *in vivo* blood ${\text{NH}}_{4}^{+}$ levels (<250 μM) the perfused gill responded with an accumulation of ammonia in the perfusate, indicating that the gill itself is capable of generating ammonia (ammoniagenesis) which is used to elevate blood ammonia levels to concentration close to 300 μM. For example, under conditions where the gill was perfused with artificial blood containing 0 mM ${\text{NH}}_{4}^{+}$ the gill generated and transported ammonia at a rate of 0.4 μmol h^−1^ ${\text{g}}_{\text{FM}}^{-1}$ into the blood. Although ammonia is generally considered toxic to organisms, our results suggest that gill tissues of *O. vulgaris* regulate extracellular ${\text{NH}}_{4}^{+}$ homeostasis to maintain blood [${\text{NH}}_{4}^{+}$] at levels of 250–300 μM. The physiological reasons for retaining a certain amount of ammonia in the blood remain speculative. Some mid-water cephalopod species accumulate ${\text{NH}}_{4}^{+}$ in exchange for Na^+^ in specialized tissues to improve buoyancy (Seibel et al., 2004). However, since these species accumulate ${\text{NH}}_{4}^{+}$ in the mM range in specialized vacuoles it is questionable in how far blood ammonium in the μM range may support buoyancy. In most animals ${\text{NH}}_{4}^{+}$ is generated through amino acid metabolism, wherein L-amino acids are first transaminated to form glutamate, which is then deaminated to ${\text{NH}}_{4}^{+}$ and α-ketoglutarate by glutamate dehydrogenase (GDH) (Wright, 1995; Nissim, 1999; Weiner and Verlander, 2013). The mammalian kidney is known to be capable of synthesizing ${\text{NH}}_{4}^{+}$ from glutamine in order to regulate pH homeostasis (Nissim, 1999; Weiner and Verlander, 2013). Here, a respiratory acidosis stimulates renal H^+^ secretion, accompanied by an increase in ${\text{HCO}}_{3}^{-}$ accumulation and ${\text{NH}}_{4}^{+}$ excretion. Since cephalopods are powerful acid-base regulators that accumulate ${\text{HCO}}_{3}^{-}$ in the mM range to compensate for an extracellular acidosis (Gutowska et al., 2010; Hu et al., 2014b) it is tempting to speculate that also here branchial ammonia production generates ${\text{HCO}}_{3}^{-}$ that can be used to regulate blood pH.

Interestingly, many aquatic organisms including fish (Lin et al., 2012), crustaceans (Fehsenfeld and Weihrauch, 2013), echinoderms (Stumpp et al., 2012; Hu et al., 2014a), and molluscs (Thomsen and Melzner, 2010) respond with increased ${\text{NH}}_{4}^{+}$ excretion rates in response to acidified conditions as well. It has been hypothesized that ${\text{NH}}_{4}^{+}$ based proton secretion might represent a universal and evolutionary ancient pathway to counter an acidosis in many organisms (Wright, 1995; Hu et al., 2014b). Applying an acidosis by reducing the pH of the artificial *octopus* blood to pH 7.2 our results clearly showed a substantial capability of the octopus gill to regulate blood pH homeostasis to maintain pH-values around 7.4–7.5. Similar to the situation in teleost fish (Evans et al., 2005) and crustaceans (Henry et al., 2012), the cephalopod gill represents an important site for extracellular pH regulation equipped with an acid-base regulation machinery located in specialized epithelial cells (Hu et al., 2011, 2014b). The present work provides a direct evidence for the coupling of extracellular pH regulation and ${\text{NH}}_{4}^{+}$ excretion during an acidosis in a molluscan system.

### Branchial ${\text{NH}}_{4}^{+}$ transport in the octopus gill

The selective soluble adenylyl cyclase (sAC) inhibitor KH7 demonstrated that the excretion of ${\text{NH}}_{4}^{+}$ across brachial epithelia is cAMP-dependent. sAC is an evolutionary ancient enzyme that is involved in ${\text{HCO}}_{3}^{-}$ sensing from cyanobacteria to humans and has been mainly associated with ion/pH regulatory epithelia (Tresguerres et al., 2011). sAC has been demonstrated to be an important regulator of primarily active ion pumps including NKA (Schmitz et al., 2014) and V-type H^+^-ATPase (Tresguerres et al., 2011). Accordingly, it can be hypothesized that also in molluscs like *O. vulgasis* sAC has inherited the evolutionary conserved role as a regulator of acid-base homeostasis. Recent advances in understanding branchial pH regulation and excretion in cephalopods have led to first models of proton and ${\text{NH}}_{4}^{+}$ secretion pathways. The cephalopod gill is equipped with acid-base transporters including NHE3, VHA, and NKA that are stimulated by a hypercapnia-induced acidosis (Hu et al., 2014b). Furthermore, a Rhesus protein (RhP) that is co-localized with NHE3 in apical membranes has been suggested to be involved in an acid-trapping mechanism of NH_3_ by protons in the semi-tubular lamellar of the squid gill (Hu et al., 2014b). Similar to the situation in decapodiform cephalopods (e.g., squid and cuttlefish) the octopus gill also expresses acid-base transporters, including NHE3, VHA, NKA, and RhP. Predominant localization of NHE3 and RhP in apical membranes and NKA in basolateral membranes suggest that also in octopus branchial epithelia, NHE3, and RhP operate in concert to promote ammonia excretion. Furthermore, the predominant cytoplasmic localization of the VHA could indicate that this transporter underlies post-translational control through membrane trafficking mechanisms and/or may be involved in an alternative, vesicular ${\text{NH}}_{4}^{+}$ excretion pathway suggested for crustacean gills and the hypodermis of *Caenorhabditis elegans* (Weihrauch et al., 2002; Henry et al., 2012; Adlimoghaddam et al., 2015, 2016). Results of these studies suggested that ${\text{NH}}_{4}^{+}$ is trapped in VHA-rich, acidified vesicles. The vesicles are then thought to be transported along the microtubules network to the apical membrane of the gill epithelium, where vesicular content is excreted by exocytosis (Weihrauch et al., 2002). Also here the establishment of gill perfusion techniques in octopus provides a new and powerful model to study pH and ammonia regulatory mechanisms in complex invertebrate systems.

### Role of different excretory organs in ammonia homeostasis

Comparisons of substrate preferences in different tissues of octopus indicated that gill, kidney (renal appendages), and liver tissues preferentially oxidize glutamate, which has been speculated to represent an important source of ${\text{NH}}_{4}^{+}$ (Hochachka and Fields, 1982). *In vitro* determinations of glutamate oxidation rates in gill and kidney tissues of *Octopus macropus* were 1.1 and 1.3 μmol h^−1^ ${\text{g}}_{\text{FM}}^{-1}$, respectively (Hochachka and Fields, 1982). Furthermore, purine catabolism has been suggested to represent another source of gill ammoniagenesis supported by the evidence of high adenosine deaminase activity in the squid gill (Hoeger et al., 1987). These biochemical data corroborate with our functional results demonstrating that the isolated octopus gill is capable of generating NH_3_/${\text{NH}}_{4}^{+}$ at rates of 4.55 ± 1.73 μmol h^−1^ ${\text{g}}_{\text{FM}}^{-1}$ under simulated *in vivo* conditions with no ${\text{NH}}_{4}^{+}$ added to the perfusion saline. Here it remains to be investigated through which pathways endogenous ammonia is exported across the basolateral membrane. In the thick ascending limb of the mammalian kidney ${\text{NH}}_{4}^{+}$ can exit the basolateral membrane via NHE4 and through a so far uncharacterized, presumably diffusive, mechanism (Weiner and Verlander, 2013). The expression data and immuno-histochemical localization of VHA, NKA, NHE3, and RhP underline the functional role of the gill in excretory processes. However, our mRNA expression data also demonstrate that among excretory organs highest expression levels for VHA, NKA, and RhP were detected in renal appendages. These organs are homologous to the vertebrate kidneys, and produce a highly acidic and ${\text{NH}}_{4}^{+}$ rich urine. Similar to gills, the renal appendages (kidneys) are a significant site of active ammonia synthesis and excretion into the renal sac (Potts, 1965; Potts and Todd, 1965; Hochachka and Fields, 1982). An acid-trapping mechanism of ${\text{NH}}_{4}^{+}$ in the highly acidified urine has been speculated, but the mechanisms of proton and proton equivalent secretion remain unknown. Furthermore, although the majority of ${\text{NH}}_{4}^{+}$ is removed after the passage of the gills, higher ammonia excretion rates during feeding (Boucher-Rodoni and Mangold, 1985) may require additional excretion capacities by the renal appendages (kidney). Thus, future studies addressing the regulatory mechanisms in different excretory organs of octopus will be important to improve our holistic understanding regarding ammonia and pH homeostasis in these highly developed molluscs.

## Conclusion

The present work highlighted the importance of a strictly regulated ${\text{NH}}_{4}^{+}$ dependent acid-base homeostasis in the cephalopod *O. vulgaris*. Our results demonstrated that these animals not only excrete ammonia into the surrounding sea water but are capable of maintaining an ammonia concentration of ~300 μM in their blood. In contrast to terrestrial vertebrates that have normal venous plasma values ranging between 5 and 40 μM ${\text{NH}}_{4}^{+}$ (Cooper and Plum, 1987) aquatic species are generally characterized by extracellular ${\text{NH}}_{4}^{+}$ concentrations in the range of 100–600 μM (Wood, 1993; Weihrauch et al., 2004; Cruz et al., 2013). In the light of our results that demonstrated a direct link between pH regulation and ${\text{NH}}_{4}^{+}$ excretion it is tempting to speculate that blood [${\text{NH}}_{4}^{+}$] in the range of 200–300 μM are essential to maintain acid-base regulatory capacities in octopus.

The present work also demonstrated that besides branchial epithelia *O. vulgaris* has additional organs that are potentially capable of mediating ${\text{NH}}_{4}^{+}$ excretion and pH regulation including renal-, and branchial heart- appendages. It can be hypothesized that coordination of excretion and acid-base regulation between different organs is controlled by endocrine mechanisms. Future studies will aim at improving this gill perfusion technique by using solutions that are closer to those seen by the gill under *in vivo* conditions using seawater (external medium) and species specific perfusion salines. These studies will include a deeper analysis of blood acid-base parameters, including changes in pH, ${\text{HCO}}_{3}^{-}$, and pCO^2^ during passage of the perfused octopus gill. Furthermore, using this perfusion technique in different cephalopod excretory organs in combination with synthesized hormone peptides (e.g., octopressin) will help to develop an *ex-vivo* model to better understand the regulatory mechanisms of excretion and pH homeostasis in cephalopods.

## Author contributions

MH, PH, YT, and DW designed and conducted experiments of the present work. YG performed molecular cloning of candidate genes from *O. vulgaris*. PS and JL conducted *in vivo* pH and ${\text{NH}}_{4}^{+}$ measurements and immunohistochemical analyses. MH, DW, and YT wrote the first draft of the manuscript and all authors contributed to the completion of the manuscript and analyses of the data.

### Conflict of interest statement

The authors declare that the research was conducted in the absence of any commercial or financial relationships that could be construed as a potential conflict of interest.
