# Supplementary material for: Perfused Gills Reveal Fundamental Principles of pH Regulation and Ammonia Homeostasis in the Cephalopod Octopus vulgaris
Source: Front Physiol. 2017 Mar 20;8:162. doi: 10.3389/fphys.2017.00162 (PMC5357659; doi:10.3389/fphys.2017.00162)
Supplement: Supplementary file 1 [file Table1.PDF]

Supplemental Table 1 Composition of Octopus saline used for perfusion experiments and native blood as well as natural seawater

Ion/amino acid	Perfusion saline (mM)	Octopus blood (mM) mean ± SD (n =6)	Natural seawater (mM)
Na^+	473.0	436.4 ± 10.5	469.6
Cl^-	521.9	649.2 ± 29.5	546.7
K^+	8.8	11.04 ± 1.2	10.2
Ca^{2+}	5.8	9.3 ± 1.3	10.3
PO_4^-	0.2	N.D.	N.D.
SO_4^-	6.5	N.D.	N.D.
HCO_3^-	2.38	2.57 ± 0.1	2.3
Pyruvate	13.6	N.D.	N.D
Fumarate	9.1	N.D.	N.D.
L-glutamine	7.5	7.42 ± 1.52	N.D.
