# Supplementary material for: Perfused Gills Reveal Fundamental Principles of pH Regulation and Ammonia Homeostasis in the Cephalopod Octopus vulgaris
Source: Front Physiol. 2017 Mar 20;8:162. doi: 10.3389/fphys.2017.00162 (PMC5357659; doi:10.3389/fphys.2017.00162)
Supplement: Supplementary file 2 [file Table2.PDF]

Supplemental Table 2 Primers used for RT-PCR

Protein name	Abbreviation	Primer sequence	Amplicon size (bp)	Reference gene ID
Na^+, K^+-ATPase	NKA	F 5’- GTGATGGGTCGTATTGCTAACT -3’<br>R 5’- CACCCTTCATCACCAGGAAATA -3’	733	JN010433.1
Sodium–hydrogen exchanger 3	NHE3	F 5’- GGTGTCACAGTGGTGTTGTA -3’<br>R 5’- CGATTTCCGTGGCTGTACTT -3’	376	XM_014934756.1
V-type proton ATPase	VHA	F 5’- TCTCGATGGGCTGAAGCTCTAA -3’<br>R 5’- CCCTTTCCTACCAGTTGGACAATT -3’	443	Bankit1370003
Rhesus (Rh) glycoprotein	RhP	F 5’- TTCTGGCAAGTTTCAACAG -3’<br>R 5’- TTCTGGATGTGGACCATGT -3’	151	BankIt1616407
**Reference genes**				
β-Actin	ACT	F 5’- CCACACTGTACCCATCTATGAAG -3’<br>R 5’- CCAGGAAGGAAGGTTGGAATAG -3’	323	AB053937.1
Ubiquitin/ribosomal protein S27a	UBQ	F 5’- CCTCCAGATCAGCAAAGATTGA -3’<br>R 5’- CCATAAACACACCAGCTCCA -3’	289	FJ617440.1

F, forward primer; R, reverse primer
