# Supplementary material for: Perfused Gills Reveal Fundamental Principles of pH Regulation and Ammonia Homeostasis in the Cephalopod Octopus vulgaris
Source: Front Physiol. 2017 Mar 20;8:162. doi: 10.3389/fphys.2017.00162 (PMC5357659; doi:10.3389/fphys.2017.00162)
Supplement: Supplementary file 3 [file Table3.PDF]

Supplemental Table 3 Primers used for qRT-PCR

Protein name	Abbreviation	Primer sequence	Amplicon size (bp)
Na^+, K^+-ATPase	NKA	F 5'- AAACTGGGATTCTATGGGCG -3'<br>R 5'- AGATCAGCCCATTGTACCAC -3'	138
Sodium–hydrogen exchanger 3	NHE3	F 5'- AAACCAGGAAGTACAGCCAC -3'<br>R 5'- GACAAATCCCGTGTGCAATG -3'	95
V-type proton ATPase	VHA	F 5'- TTCTATGAACGAGCTGGCAG -3'<br>R 5'- GCAGAAGTAACAGGGTCAGAG -3'	119
Rhesus glycoprotein	RhP	F 5'- AACAGCGCCGGTGCAGCAA -3'<br>R 5'- TGTGCAGGCGCAGAGTGAGAGA -3'	78
**Reference genes**			
β-Actin	ACT	F 5'- TCCTTCCTGGGTATGGAATCTGCTGGTATC -3'<br>R 5'- CCGGACAAGACAGTGTTGGCGTACAAATC -3'	110
Ubiquitin/ribosomal protein S27a	UBQ	F 5'- CAAGGCAAAGATTCAAGATAAGGAG -3'<br>R 5'- AGACGAAGGACCAAATGAAGG -3'	141

F, forward primer; R, reverse primer
