# Supplementary material for: Perfused Gills Reveal Fundamental Principles of pH Regulation and Ammonia Homeostasis in the Cephalopod Octopus vulgaris
Source: Front Physiol. 2017 Mar 20;8:162. doi: 10.3389/fphys.2017.00162 (PMC5357659; doi:10.3389/fphys.2017.00162)
Supplement: Supplementary file 4 [file DataSheet1.PDF]

Supplemental Figure S1

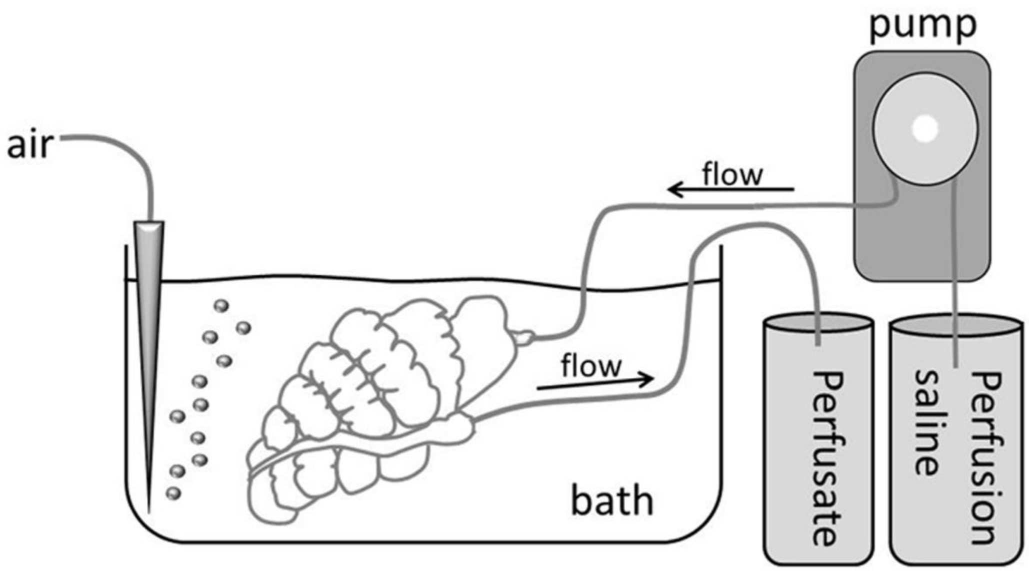

Figure S1 Schematic illustration of the perfusion setup
The isolated gill was perfused inside a 50 ml chamber using a peristaltic pump connected to the afferent and efferent vessel of the gill. One vessel contained the perfusion saline that was pumped at a rate of 12 ml h^{-1} through the gill and the perfusate was collected in a second vessel. The gill was aerated with air using a plastic tube that was inserted into the prefusion bath.
