# Supplementary material for: Perfused Gills Reveal Fundamental Principles of pH Regulation and Ammonia Homeostasis in the Cephalopod Octopus vulgaris
Source: Front Physiol. 2017 Mar 20;8:162. doi: 10.3389/fphys.2017.00162 (PMC5357659; doi:10.3389/fphys.2017.00162)
Supplement: Supplementary file 5 [file DataSheet2.PDF]

Supplemental Figure S2

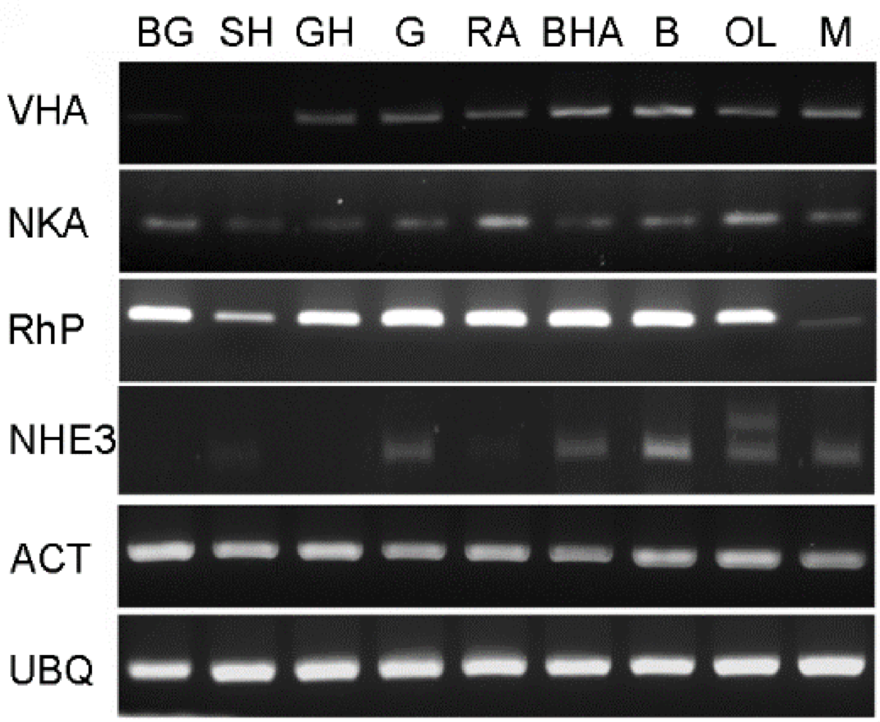

Figure S2 mRNA expressions of acid-base relevant genes in various tissues of *Octopus vulgaris*. Agarose gel of PCR-amplified cDNAs for V-type proton ATPase (VHA), Na^+/K^+-ATPase (NKA), Rhesus glycoprotein (RhP), Na^+/H^+ exchanger 3 (NHE3) with two reference genes β-Actin (ACT) and ubiquitin/ribosomal protein S27a (UBQ) as the internal control (primers set as listed in Supplemental Table 2)
