# Supplementary material for: Perfused Gills Reveal Fundamental Principles of pH Regulation and Ammonia Homeostasis in the Cephalopod Octopus vulgaris
Source: Front Physiol. 2017 Mar 20;8:162. doi: 10.3389/fphys.2017.00162 (PMC5357659; doi:10.3389/fphys.2017.00162)
Supplement: Supplementary file 6 [file DataSheet3.PDF]

# Supplemental Figure S3

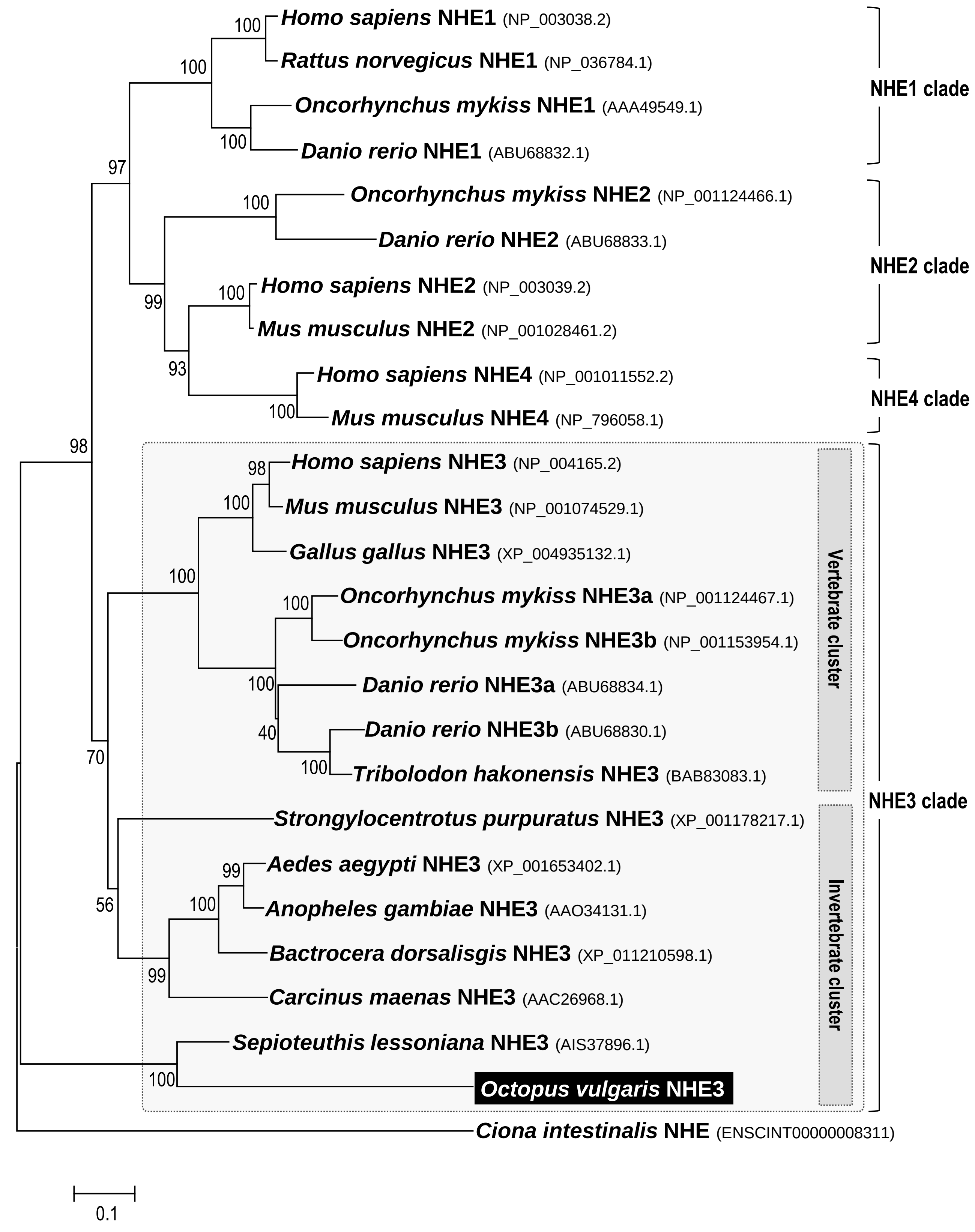


**Figure S3 Routed phylogenetic tree for *Octopus vulgaris* Na⁺/H⁺-exchanger (NHE) 1-4 isoforms.** Phylogenetic comparisons are based on amino acid sequences from NHE clades 1–4. Numbers indicate bootstrap values and accession numbers for the sequences are provided along with species names.
