# Supplementary material for: Perfused Gills Reveal Fundamental Principles of pH Regulation and Ammonia Homeostasis in the Cephalopod Octopus vulgaris
Source: Front Physiol. 2017 Mar 20;8:162. doi: 10.3389/fphys.2017.00162 (PMC5357659; doi:10.3389/fphys.2017.00162)
Supplement: Supplementary file 7 [file DataSheet4.PDF]

Supplementary Information for Material and Methods

Determination of blood pH and NH_4^+ concentrations

Determination of pH_e was performed in 500 µl samples at 25°C. NH_4^+ determinations were conducted as previously described using an orthophthaldialdehyde-based method to fluometrically determine NH_4^+ concentrations in blood samples. Excitation and emission wavelength of 360 and 422 nm were used and samples were measured in triplicates using a microplate reader (Molecular Device, Spectra Max, M5). This method has been demonstrated to be suitable for NH_4^+ determinations in blood samples as it is specific to NH_4^+ and insensitive to amino acids and proteins. Due to a pKa of ca. 9.3 for ammonia the majority >90% is found in the ionic form at a reaction pH close to neutral pH.

Molecular cloning

Obtained PCR products were sub-cloned into a pGEM-T Easy vector (Promega, Madison, WI, USA), and the nucleotide sequences were determined with an ABI 3730XL DNA Analyzer (Applied Biosystems, Applied Biosystems, Warrington, United Kingdom). Sequence analysis was conducted with the BLASTx program (NCBI). Moreover, in order to verify the membership of cloned NHE paralogues in octopus within the core family of NHE proteins, the deduced amino acid sequences of cloned NHE was aligned using ClustalW together with other orthologs collected from the NCBI and ENSEMBL databases. The obtained sequences were then subjected to phylogenetic inferences using the neighbor-joining (NJ) method (p-distance) (Supplemental Figure S3). Ten thousand bootstrap replicate analyses were carried out with Mega5.0.

Preparation of mRNA

Different tissues were excised out of the animal and subsequently homogenized in Trizol reagent (Invitrogen, Carlsbad, CA, USA) using a Tissue lyser (Qiagen). Total RNA was extracted from the aqueous phase after addition of chloroform to Trizol homogenates and purified by addition of isopropanol. DNA contamination was removed with DNase I (Promega). The mRNA for the RT-PCR was obtained with a QuickPrep Micro mRNA Purification Kit (Amersham Pharmacia, Piscataway, NJ, USA) according to the supplier protocol. The amount of mRNA was determined by spectrophotometry (ND-2000, NanoDrop Technol, Wilmington, DE), and the mRNA quality was checked by running electrophoresis. All mRNA pellets were stored at −80°C.

Real-time quantitative PCR (qPCR)

Specific primers for all genes were designed using Primer Premier software (vers. 5.0; PREMIER Biosoft International, Palo Alto, CA). PCR assays contained 40 ng of cDNA, 50 nM of each primer, and the LightCycler® 480 SYBR Green I Master (Roche) in a final volume of 10 µl. All qPCR reactions were performed as follows: After denaturation with 95°C for 5 min, 45 cycles of 95°C for 10 sec, 60°C for 10 sec and 72°C for 10 sec were processed, followed by 1 cycle of 95°C for 5 sec and 65°C for 1 min (the standard annealing temperature of all primers). PCR products were subjected to a melting-curve analysis, and representative samples were electrophoresed to verify that only a single product was present. All primer pairs used in this PCR had efficiencies >96%. Control reactions were conducted with nuclease-free water to determine levels of background. Additionally, no PCR product was obtained by using DNase I treated RNA samples as template demonstrating the

success of the DNase I treatment. The standard curve of each gene was confirmed to be in a linear range with Ubiquitin/ribosomal protein S27a (*UBQ*) and actin (*ACT*) as reference genes. Tested genes were normalized to the geometric mean of these two reference genes.
